# Correlation between pancreatic cancer and metabolic syndrome: A systematic review and meta-analysis

**DOI:** 10.3389/fendo.2023.1116582

**Published:** 2023-04-11

**Authors:** Lei Zhong, Jifeng Liu, Shuo Liu, Guang Tan

**Affiliations:** ^1^ Department of General Surgery, The First Affiliated Hospital of Dalian Medical University, Dalian, China; ^2^ Department of Endocrinology and Metabolic Diseases, The First Affiliated Hospital of Dalian Medical University, Dalian, China

**Keywords:** metabolic syndrome, pancreatic cancer, metabolic component, meta-analysis, pancreas

## Abstract

**Objective:**

Pancreatic cancer is a globally frequent cause of death, which can be caused by many factors. This meta-analysis was performed to assess the correlation between pancreatic cancer and metabolic syndrome (MetS).

**Methods:**

Publications were identified by searching PubMed, EMBASE, and the Cochrane Library for studies published until November 2022. Case-control and cohort studies published in English that provided information on the odds ratio (OR), relative risk (RR), or hazard ratio (HR) of metabolic syndrome and pancreatic cancer were included in the meta-analysis. Two researchers separately retrieved the core data from the included Random effects meta-analysis was conducted to summarize the findings. Results were presented as relative risk (RR) and 95% confidence interval (CI).

**Results:**

MetS showed a strong association with an increased risk of developing pancreatic cancer (RR1.34, 95% CI1.23–1.46, *P*<0.001), and gender differences were also observed (men: RR 1.26, 95% CI 1.03–1.54, *P*=0.022; women: RR 1.64, 95% CI 1.41–1.90, *P*< 0.001). Moreover, an increased risk of developing pancreatic cancer was strongly linked to hypertension, poor high-density lipoprotein cholesterol, and hyperglycemia (hypertension: RR 1.10 CI 1.01–1.19, *P*=0.027; low high-density lipoprotein cholesterol: RR 1.24 CI 1.11–1.38, *P*<0.001; hyperglycemia: RR 1.55, CI 1.42–1.70, *P*< 0.001). However, pancreatic cancer was independent of obesity and hypertriglyceridemia (obesity: RR 1.13 CI 0.96–1.32, *P*=0.151, hypertriglyceridemia: RR 0.96, CI 0.87–1.07, *P*=0.486).

**Conclusions:**

Although further prospective studies are required for confirmation, this meta-analysis indicated a strong relationship between MetS and pancreatic cancer. Regardless of gender, a greater risk of pancreatic cancer existed in people with MetS. Patients with MetS were more likely to develop pancreatic cancer, regardless of gender. Hypertension, hyperglycemia, and low HDL-c levels may largely account for this association. Further, the prevalence of pancreatic cancer was independent of obesity and hypertriglyceridemia.

**Systematic review registration:**

https://www.crd.york.ac.uk/prospero/, identifier CRD42022368980.

## Introduction

Pancreatic cancer (PC) is a common malignant tumor type with the 12^th^-highest incidence rate among all malignant tumors (1). PC has a dismal prognosis, with a general five-year relative survival rate of 10%, and it is the fourth and sixth most widely occurring common cause of cancer-related mortality in China and the United States, respectively (2, 3).The risk factors are unclear, and PC may develop in patients with a family history of cancer as well as those who smoke, drink alcohol, are obese, or have diabetes (4).

The metabolic syndrome (MetS) has attracted considerable attention with regard to its association with cardiovascular risk factors, first proposed in 1988 (5).Dyslipidemia, central obesity, poor glucose tolerance, insulin resistance, type 2 diabetes, and hyperinsulinemia are some abnormal metabolic parameters characterizing MetS (6). These parameters are typically assessed using the following indicators: blood pressure, fasting plasma glucose level, waist size, high-density lipoprotein cholesterol (HDL-c) levels, and triglyceride level (7). MetS or its components may be linked to numerous malignancies, including breast, colorectal, endometrial, and gastric cancer (8–11). MetS were also investigated as a potential PC risk factor. It was observed that in the general public, it was strongly linked to an elevated risk of developing PC (12). Previously, the number of MetS components and the probability of developing PC showed a strong correlation (13). The risk of PC varied among people with MetS, with the presence of four or five metabolic components being linked to the highest risk (14). However, a Japanese study found that only women with two or more metabolic components showed an elevated risk of PC (15). A subsequent prospective study, including over 580,000 people, also supported these findings (16). However, several shortcomings of these studies, including insufficient sample size, lack of ethnic/racial heterogeneity, and an inadequate assessment of confounders and/or reverse causality, resulted in contradictory findings.

Several studies have demonstrated that various aspects of MetS, such as obesity and type 2 diabetes, can increase the risk of PC (16–18). However, it is unclear which aspect of MetS is most strongly associated with PC and whether gender influences the effect of MetS on PC. The effects of MetS as a risk factor on PC were thoroughly reviewed and subjected to a meta-analysis. Furthermore, sub-analyses based on gender were conducted.

## Methods and materials

### Search strategy

PubMed, Embase, and the Cochrane Library databases were systematically searched for pertinent studies that were published between the creation of the database and November 1, 2022. The following search terms were used: (‘pancreatic carcinoma’ OR ‘pancreatic cancer’ OR ‘pancreatic adenocarcinoma’ OR ‘pancreatic neoplasms’) AND (‘metabolic syndrome’ OR ‘Metabolic X Syndrome’ OR ‘Dysmetabolic Syndrome X’ OR ‘MetS’). Furthermore, the reference lists of qualified articles were visually examined for any additional pertinent studies.

### Selection criteria

Based on the inclusion and exclusion criteria listed below, two researchers screened the retrieved publications independently, and discrepancies were settled by consensus. The following inclusion criteria were applied: (1) the publication that was written in English and was a cohort study or a case-control study; (2) data on the relative risk (RR), odds ratio (OR), or hazard ratio (HR) with a 95% confidence interval (95%CI) were available; (3) when multiple publications were produced from the same data, only the most comprehensive paper was selected.

Exclusion criteria for this study were as follows: (1) letters, case reports, reviews, expert opinions, or editorials were excluded; (2) excluded if they lacked critical data; (3) excluded if they failed to mention MetS and diagnostic criteria for PC explicitly; or (4) they were duplicates of other studies. Additionally, case-control studies were excluded from the meta-analysis but included in the systematic review.

### Quality assessment

The Newcastle-Ottawa Scale (NOS) for quality evaluation of cohort studies and case-control studies was used to independently evaluate study quality (19). The NOS comprises eight components assigned to three groups based on selection, comparability, and research type exposure (case-control studies) or outcome (cohort studies). For each issue, a number of response alternatives were offered. A star system was employed to provide a semi-quantitative evaluation of the quality of the study. The highest-quality studies yielded a maximum of one star for each item, with the exception of the comparability item, which makes two stars. The NOS stars range between zero to nine. We discussed any disagreements until an agreement was reached. After examination, it was concluded that each study under investigation was of moderate to high quality.

### Data extraction

The names of the first authors, the year the study was published, the country where the investigation was done, the duration of follow-up, the total number of patients, and the criteria for the definition of MetS were all retrieved separately by the two researchers for each study that was accessible. Using the most adjusted model, we derived the pooled risk estimates and associated 95%CIs. A discussion was used to settle any disagreements. To assess the effects of MetS components on the risk of developing PC, risk estimates were also gathered for each individual MetS component.

### Statistical analyses

Using pertinent risk estimations, the relative risks (RR), hazard ratios (HRs), incident rate ratios, standardized incidence ratios (SIRs), and their 95%CIs were employed to evaluate the relationship between MetS and PC risk. From the multivariable models of the original studies, adjusted risk estimates were generated. Additionally, we assessed how each component of the metabolic syndrome affected the risk of PC on an individual basis. Sensitivity analysis was also carried out to test whether any of the studies had shown a significant impact on the outcome. Using the random-effects model, the outcomes of the retrieved papers were combined. In order to evaluate the statistical heterogeneity across studies, the I^2^ statistic was used. Low, moderate, and high levels of heterogeneity were estimated to be 25%, 50%, and 75%, respectively. To assess publication bias, the Egger test and funnel plotting were performed. When at least ten original publications were included, a P value < 0.05 showed publication bias. STATA (version 16.0) was used for conducting all analyses, and statistical significance was established at P< 0.05.

## Results

### Search results

**Figure 1:**
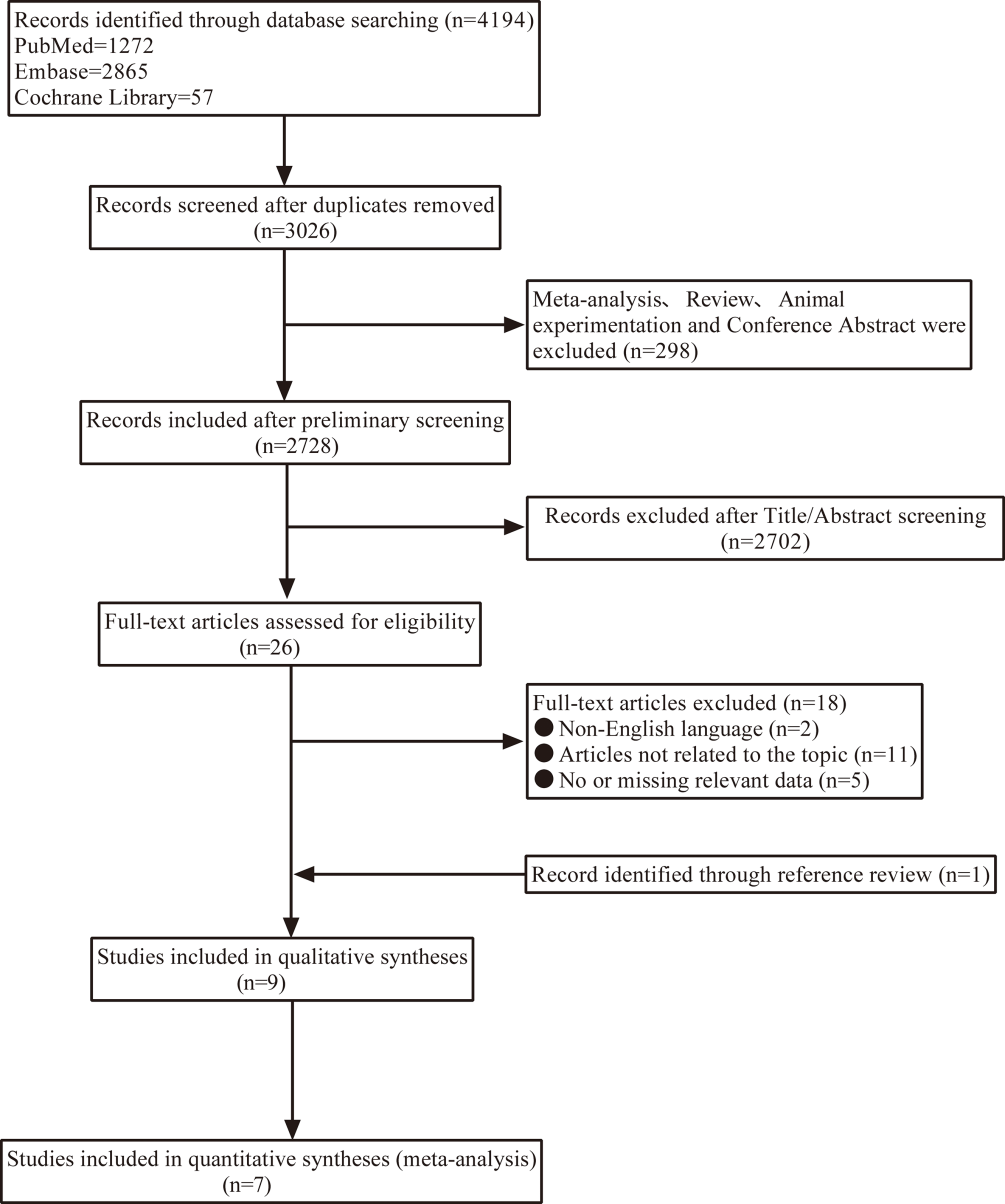
Flowchart for screening the literature.

**Figure 1** displays a flow chart that illustrates the literature screening process. In total, 4,194 articles were retrieved from databases. Nine publications (12–15, 20–24), comprising two case-control studies (13, 23) and seven cohort studies (12, 14, 15, 20–22, 24), were considered in the systematic review. All the duplicate studies and those studies that failed to meet the inclusion criteria were eliminated. Meta-analysis was performed on all cohort studies (**Figure 1**).

### Characteristics of included studies

A complete summary of the fundamental characteristics of each study that was included in this research is provided in **Table 1**. The study comprises research published between 2008 and 2022, and their quality scores, on average, were 7.2 stars. The median follow-up period per a study in the included literature ranged from 2.7 (Russo et al.) to 10.2 (Manami Inoue et al.) years. The adjusted analyses showed varied potential confounding factors (risk factors), including a maximum of 10 (21, 24) and a minimum of 5 confounders (15). In addition, only four studies reported an association between high blood glucose, blood pressure, triglyceride levels, and HDL-c levels with PC (12, 14, 22, 24). In comparison, five studies reported an association of obesity with PC (12, 14, 21, 22, 24).

**Table 1:** Characteristics of the studies included in the quantitative and qualitative review.

Author	Year	Country	Study Type	Age (range or mean)	MetS criteria	Follow-up	Sample size	No. of cases	Quality assessment
Antonio Russo (20)	2008	Italy	Cohort	≥40	Pharmacological definition	median follow-up 2.7 years	16,677	43	6
Manami Inoue (15)	2009	Japan	Cohort	M:56.5 ± 8.2 F:55.5 ± 8.1	AHA	average follow-up 10.2 years	27,724	65	6
Valentina Rosato (13).	2011	Italy	Case control	34-80	AHA	17years	978	21	6
Bin Xia (12).	2020	China	Cohort	MetS (+):58.1 MetS (-):55.8	IDF	MetS (+):6.5 years(1.3) MetS (-):6.6 years(1.2)	475,078	565	8
Sung Keun Park (14)	2020	South Korea	Cohort	MetS (+):60.3 ± 9.1 MetS (-):56.92 ± 8.4	IDF	4years	222,838	381	8
HyeSoo Chung (21)	2021	South Korea	Cohort	MetS (+):60 ± 9 MetS(-):59.3 ± 8.7	IDF	median follow-up 6.1 years	347,434	886	7
Joo-Hyun Park (22)	2022	South Korea	Cohort	48.9	IDF	median follow-up 5.1 years	8,203,492	8010	8
Joseph A (24)	2022	French	Cohort	MetS (+):58.40 ± 7.61 MetS(-):55.48 ± 8.15	NCEP-ATPIII	median follow-up 7.1 years	366,494	478	8
Tomàs López-Jiménez (23)	2022	Spain	Case control	≥40	AHA	11years	183,284	1996	8

AHA, American Heart Association;

IDF, International Diabetes Federation;

NCEP-ATP III, National Cholesterol Education Program Adult Treatment Panel III.

### Meta-analysis results

**Figure 2:**
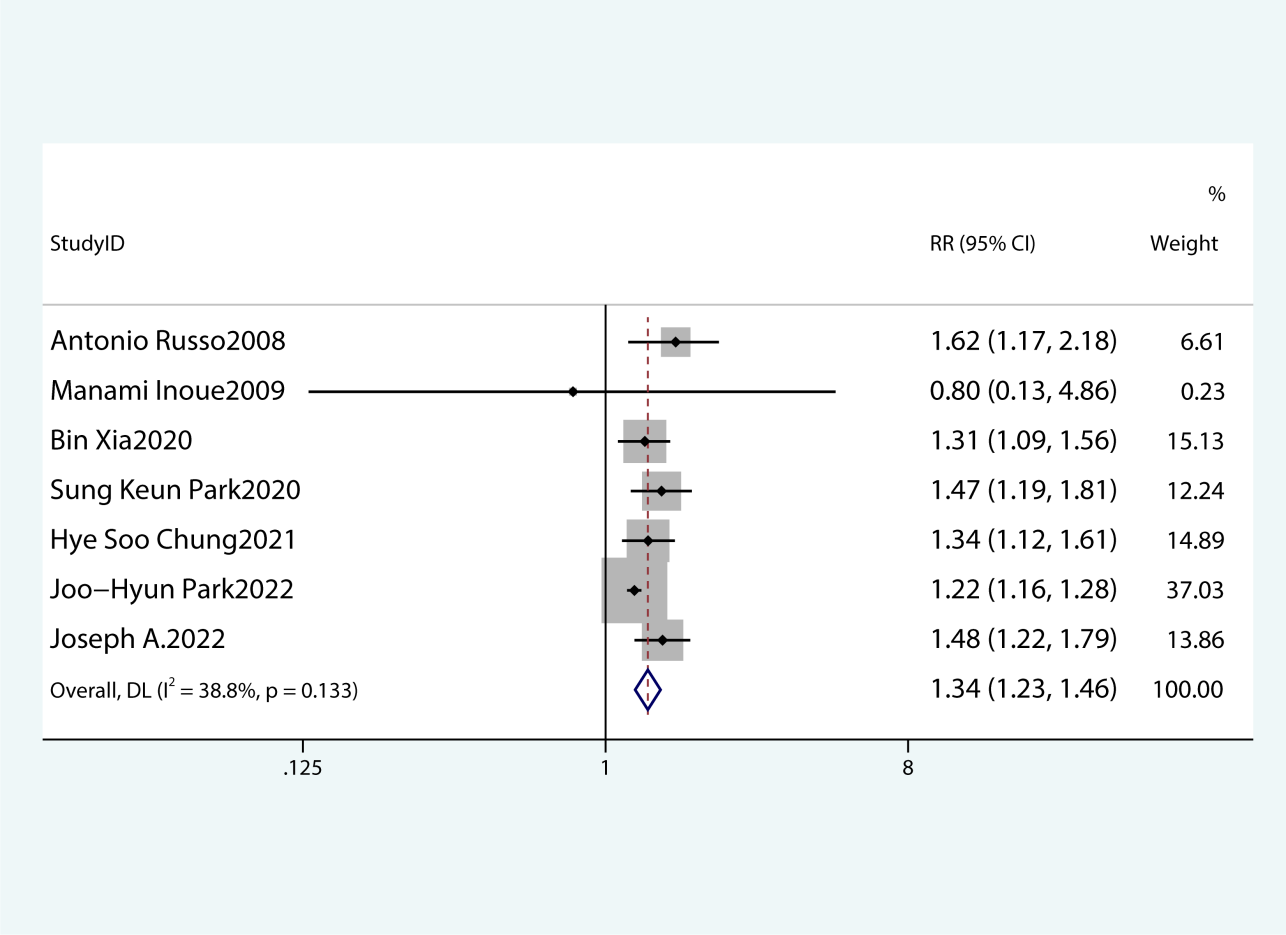
Meta-analysis of studies on the correlation of metabolic syndrome with pancreatic cancer;.

**Figure 3:**
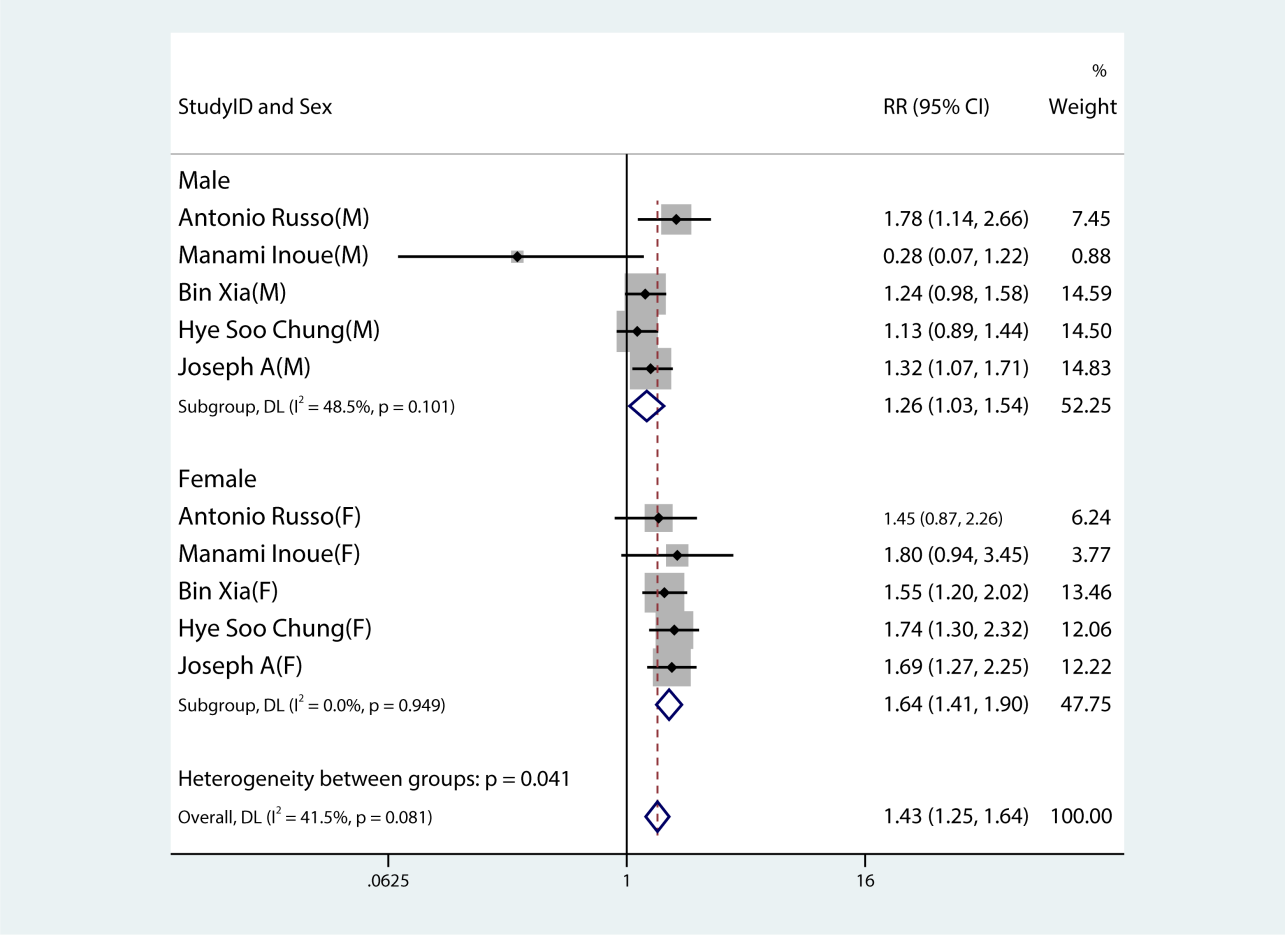
Forest plot demonstrating the association between the metabolic syndrome and the risk of developing pancreatic cancer in both males and females.

**Figure 4:**
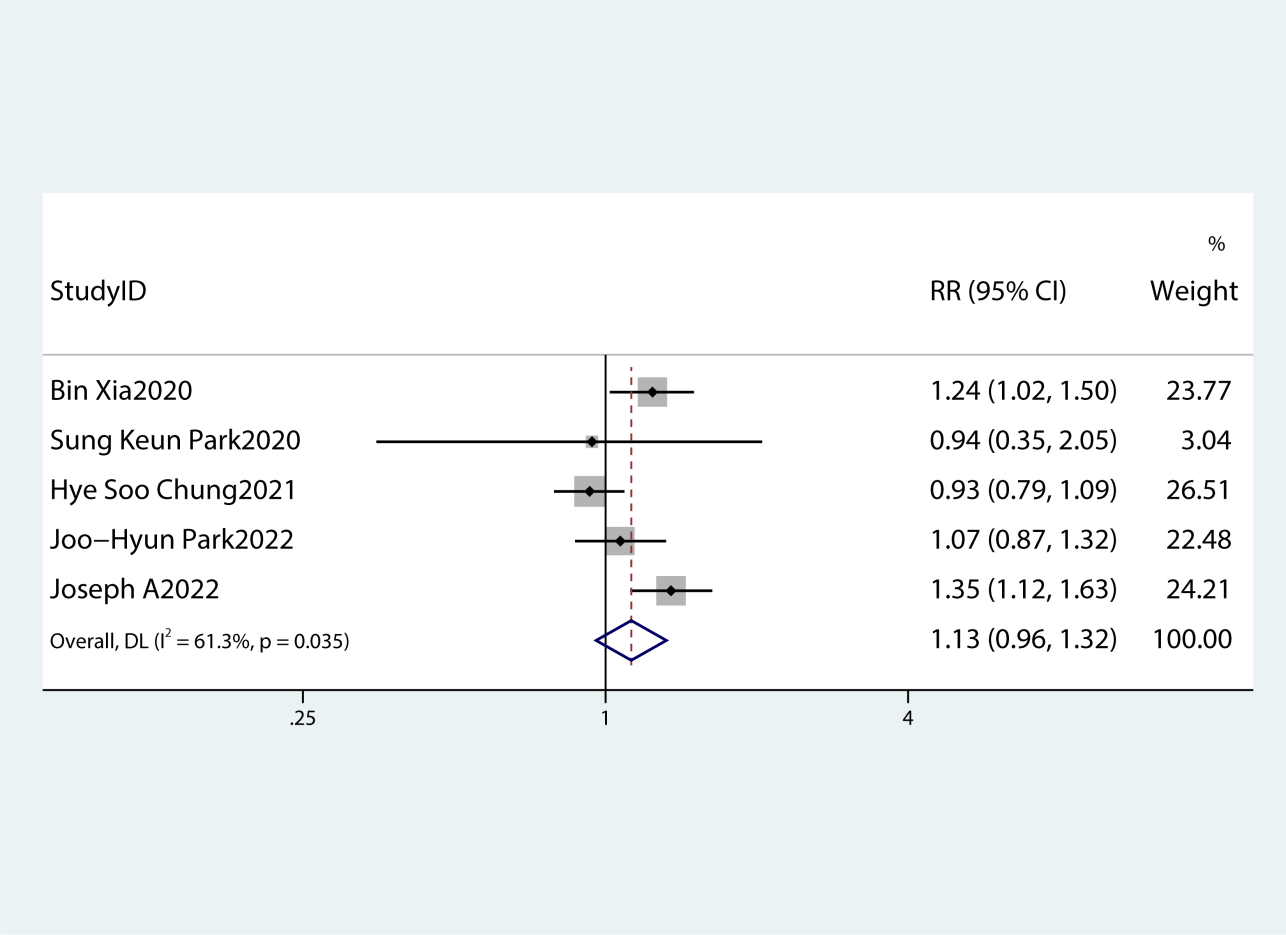
A forest plot demonstrating the relationship between obesity and the risk of pancreatic cancer.

**Figure 5:**
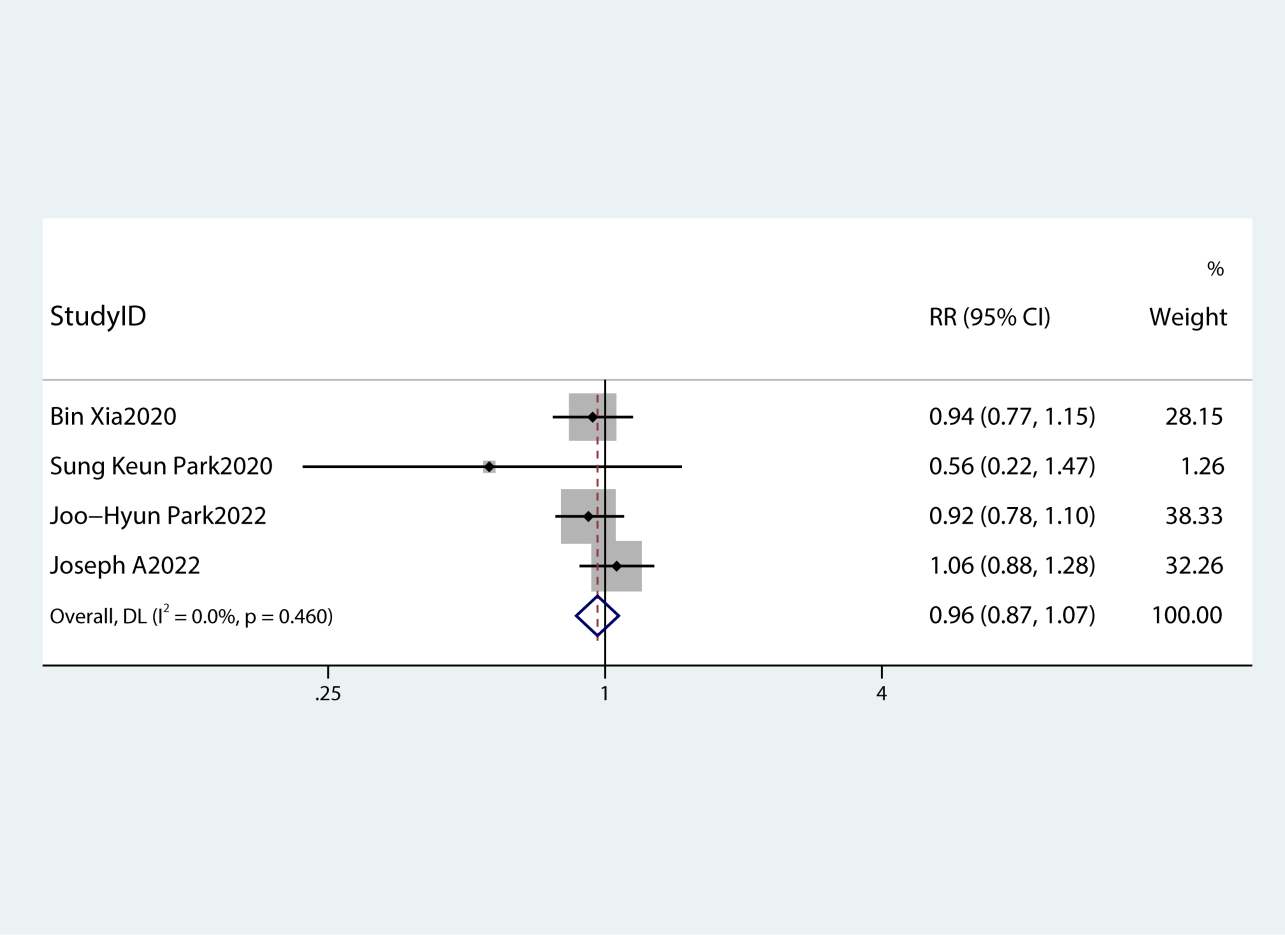
A forest plot demonstrating the relationship between hypertriglyceridemia and the risk of pancreatic cancer.

**Figure 6:**
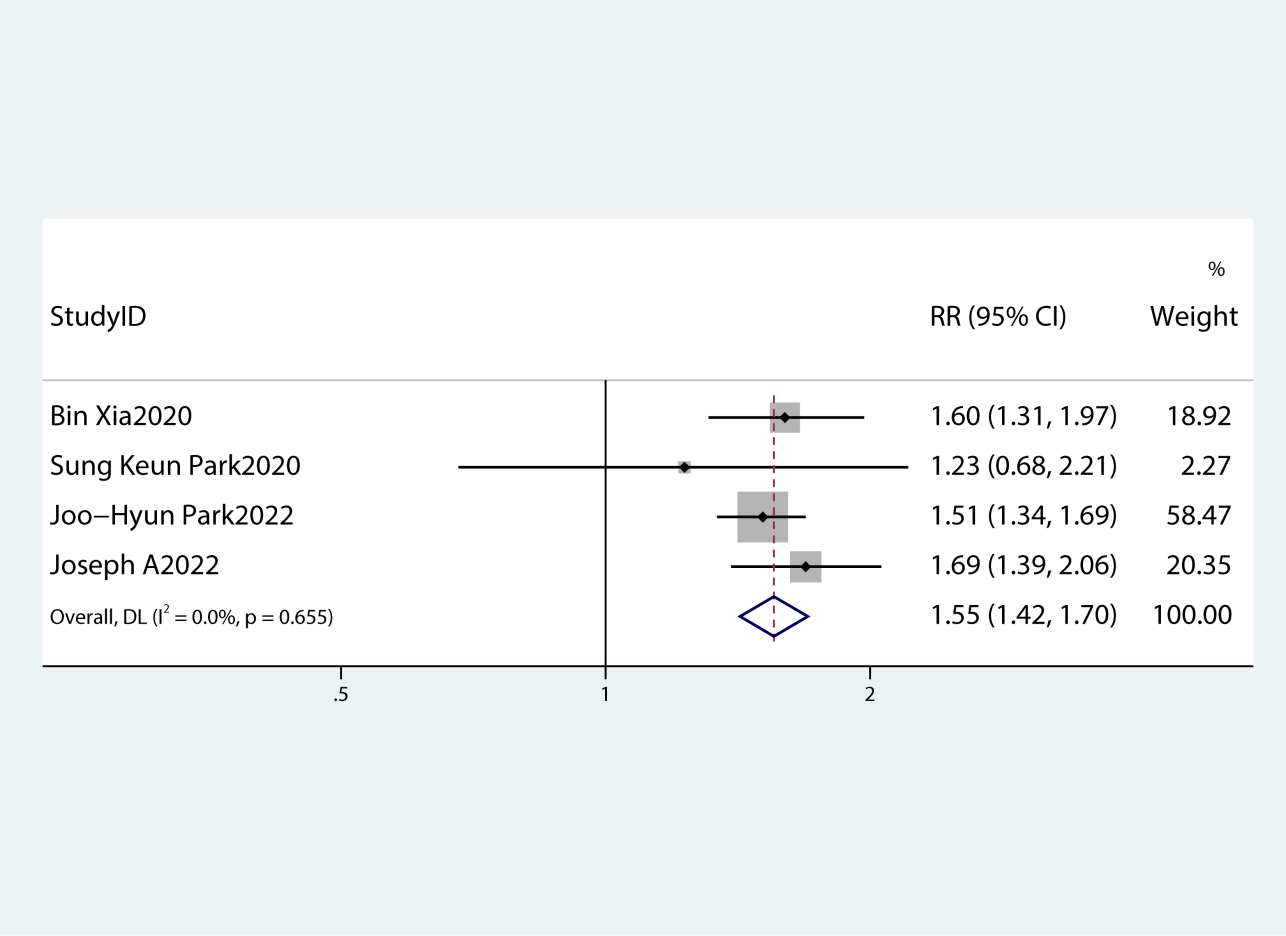
A forest plot demonstrating the relationship between hyperglycemia and the risk of pancreatic cancer.

**Figure 7:**
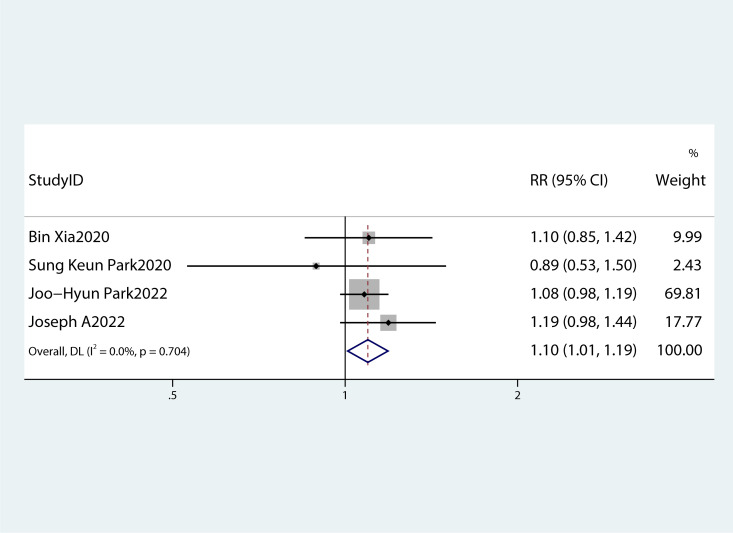
A forest plot demonstrating the relationship between hypertension and the risk of pancreatic cancer.

**Figure 8:**
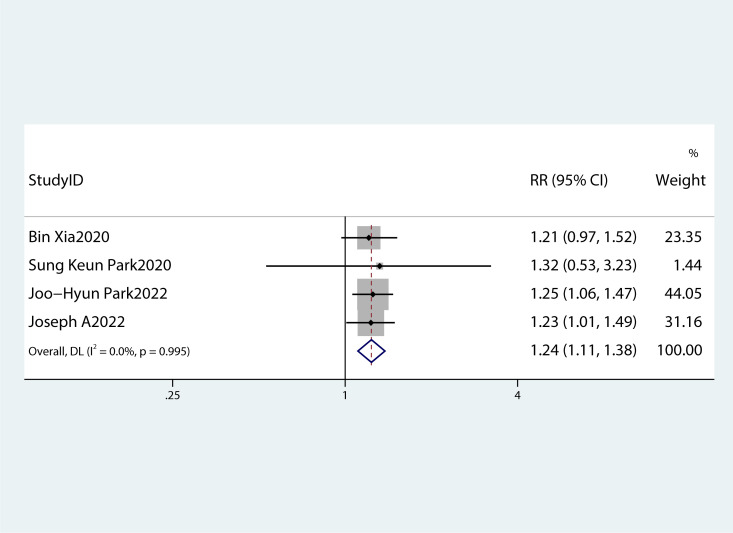
A forest plot demonstrating the relationship between low HDL-c levels and the risk of pancreatic cancer.

**Figures 2**–**8** show forest plots for the PC and MetS meta-analysis. In comparison with non-MetS individuals, patients having MetS had a greater probability of getting PC (RR 1.34, 95%CI 1.23–1.46, *P*<0.0001, I^2^ = 38.8%) (**Figure 2**). For the subgroup analysis of the prevalence of PC in MetS patients, the study population was divided into male and female groups. It was observed that the prevalence of PC was remarkably higher in males and females with MetS than among non-MetS patients. Among MetS patients, females were more likely to develop PC than males (male: RR 1.26, 95%CI 1.03–1.54, *P*=0.022; females: RR 1.64, 95%CI 1.41–1.90, *P*< 0.001) (**Figure 3**).

**Figure 9:**
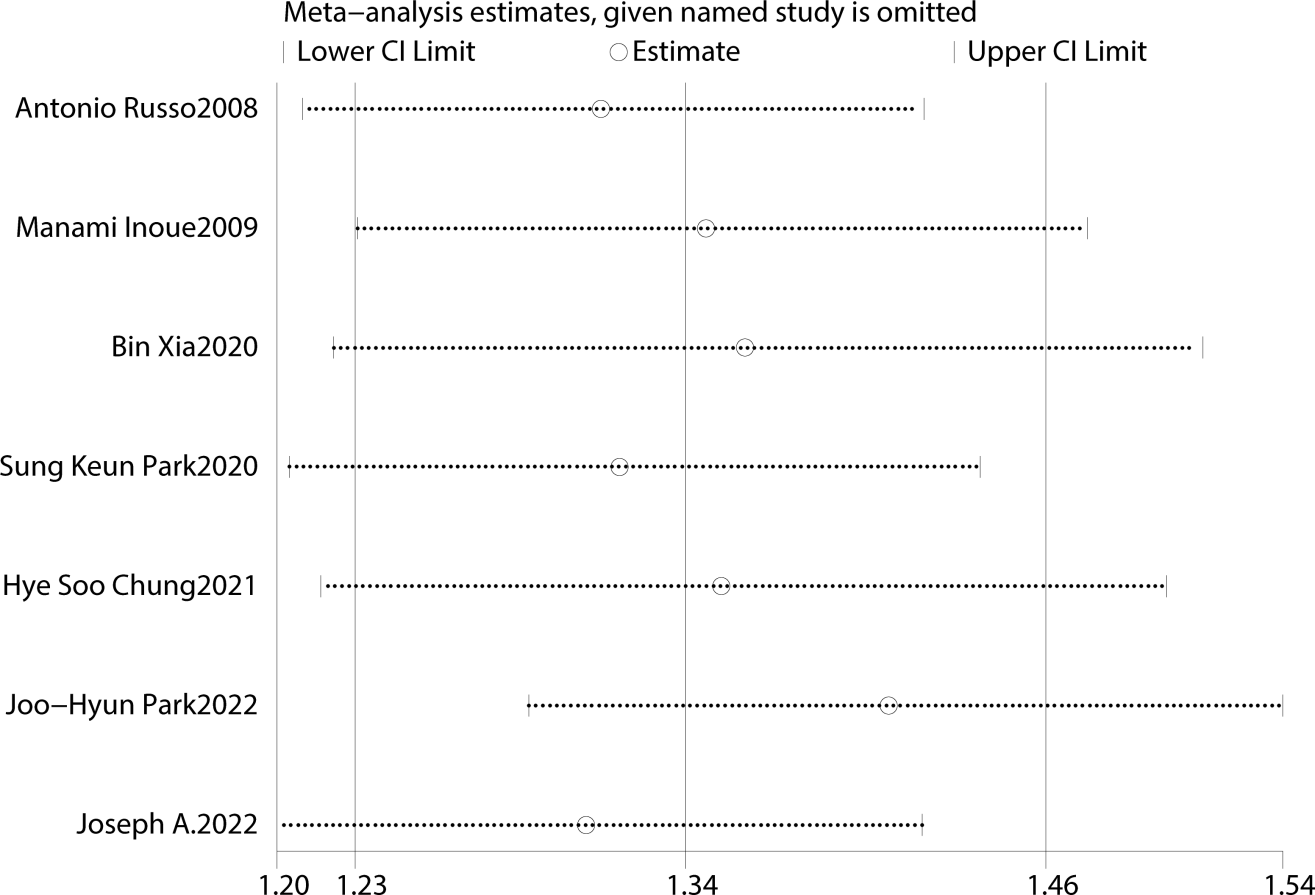
Sensitivity analyses for pancreatic cancer;.

**Table 2** summarizes the diagnostic criteria for every single component of MetS present in each study, and **Table 3** lists the numerous types of diagnostic criteria for MetS. These findings demonstrated that the risk of PC was not correlated with obesity or hypertriglyceridemia (obesity: RR 1.13, 95%CI 0.96–1.32, *P*=0.151; hypertriglyceridemia: RR 0.96, 95%CI 0.87–1.07,*P*=0.486) (**Figures 4**, **5**).While hyperglycemia, hypertension and low HDL-c increased the risk of PC (hyperglycemia: RR 1.55,95%CI 1.42–1.70, *P*< 0.001; hypertension: RR 1.10, 95%CI 1.01–1.19, *P*=0.027; low HDL-c: RR 1.24, 95%CI 1.11–1.38, *P*< 0.001) (**Figures 6**–**8**). The results of the sensitivity analysis showed that the link between MetS and the risk of PC was unaffected noticeably due to the lack of any studies (**Figure 9**).

**Table 2:** Diagnostic criteria for any single component of metabolic syndrome in each study.

Author	Year	hypertension	hyperglycemia	obesity	hypertriglyceridemia	Low HDL-c
Antonio Russo (20)	2008	Use of drugs for hypertension	Use of drugs for diabetes	-	-	Use of drugs for hypercholesterolemia
Manami Inoue (15)	2009	BP≥130/85 mmHg and/or use of antihypertensive agents	glucose ≥ 5.55 mmol/l (100 mg/dl) fasting or ≥ 7.77 mmol/l (140 mg/dl) non- fasting	BMI ≥ 25 kg/m^2^	high serum triglycerides ≥ 1.69 mmol/l (150 mg/dl)	low HDL-c< 1.03 mmol/l (40 mg/dl) for men and <1.29 mmol/l (50 mg/dl) for women
Bin Xia (12)	2020	systolic ≥ 130 mmHg or diastolic ≥ 85 mmHg or treatment of previously diagnosed hypertension	FPG ≥ 100 mg/dLor previously diagnosed type 2 diabetes	BMI > 30 kg/m^2^	TGlevels ≥ 0.7 mmol/L (150 mg/dL) or currently on medications for hypertriglyceridaemia	HDL-c< 0.9 mmol/L (40 mg/dL) for men and < 1.29 mmol/L (50 mg/dL) in women or specific treatment for previously detected reduced HDL -c.
Sung Keun Park (14)	2020	BP ≥130/85 mm Hg	FPG ≥ 100 mg/dL	WC≥90 cm in men and ≥85 cm in women	TG levels ≥150 mg/dL	HDL-c< 40 mg/dL for men and < 50 mg/dL for women
Hye Soo Chung (21)	2021	BP ≥130/85 mmHg or the use of antihypertensive agents	FPG≥5.6 mmol/L (100 mg/dL) or useofan antidiabetic drug	BMI is ≥25 kg/m^2^	serum triglyceride levels ≥1.7 mmol/L (≥150 mg/dL) or the current use of lipid-lowering agents	HDL-c <1.0 mmol/L (40 mg/dL) in men or<1.3 mmol/L (50 mg/dL) in women or the current use of lipid-lowering agents
Joo-Hyun Park (22)	2022	systolic ≥130 or diastolic ≥80 mmHg or the use of antihypertensive agents	FPG≥100 mg/dL or the use of an antidiabetic drug	WC≥90 cm in men and ≥85 cm in women	TG levels ≥150 mg/dL or the use of a relevant drug	HDL-C <40 mg/dL for men and <50 mg/dL for womenor the use of a relevant drug
JosephA (24)	2022	systolic ≥ 130 mmHg and diastolic ≥ 85 mmHg, or previously diagnosed high BP, or regular use of BP-lowering medication.	HbA1c≥ 5.7%, regardless of diabetes status.	WC≥102 cm in men or ≥88 cmin women	triglycerides were considered elevated if measured at ≥1.7 mmol/L	reduced HDL was defined as ≤1.03 mmol/L in men and ≤1.29 mmol/L in women, or regular use of cholesterol-lowering medication

BP, blood pressure; FPG, fasting plasma glucose; BMI, body mass index; WC, waist circumference; TG, plasma triglyceride; HDL-c, high-density lipoprotein cholesterol.-, It means that the diagnostic criteria for this component of metabolic syndrome are notprovided in the article.

**Table 3:** Different Criteria for MetS Diagnosis.

MetS Diagnosis Criterion	Details
Pharmacological definition	Patients who are also taking medicine for high cholesterol, high blood pressure, and diabetes
NCEP-ATP III	(1) WC≥102 cm in men and ≥88 cmin women; (2) TG≥1.7 mmol/L; (3) HDL-c ≤ 1.03mmol/L in men and ≤1.29 mmol/L in women (4) BP≥130/85 mmHg; (5) FPG≥6.1 mmol/L; ≥3 above components can be diagnosed as MetS.
IDF	(1) central obesity(WC ≥ 90 cm and ≥ 80 cm in Asians, with other values for other ethnicities; or BMI > 30 kg/m2); (2) TGlevels ≥ 0.7 mmol/L (150 mg/dL); (3) HDL-c< 0.9 mmol/L (40 mg/dL) for men and < 1.29 mmol/L (50 mg/dL) in women or specific treatment for previously detected reduced HDL -c; (4) systolic ≥ 130 mmHg or diastolic ≥ 85 mmHg or treatment of previously diagnosed hypertension; (5) FPG ≥ 100 mg/dLor previously diagnosed type 2 diabetes; central obesity plus any two of the above four factors can be diagnosed as MetS.
AHA	(1) FPG ≥ 100 mg/dL or receiving drug therapy for hyperglycemia; (2) BP ≥ 130/85 mmHg or receiving drug therapy for hypertension; (3) TG ≥ 150 mg/dL or receiving drug therapy for hypertriglyceridemia; (4) HDL-c < 40 mg/dL in men or < 50 mg/dL in women or receiving drug therapy for reduced HDL-C; (5) WC≥90 cm in men or ≥80 cmin women; ≥3 above components can be diagnosed as MetS.

NCEP-ATP III, National Cholesterol Education Program Adult TreatmentPanel III;

IDF, International Diabetes Federation;

AHA, American Heart Association;

BP, blood pressure;

FPG, fasting plasma glucose;

BMI, body mass index;

WC, waist circumference;

TG, plasma triglyceride;

HDL-c, high-density lipoprotein cholesterol.

## Discussion

Among the components of MetS, dyslipidemia, hypertension, diabetes, and obesity-related biological processes are closely related to one another and increase the risk of developing numerous diseases. The strongest risk factor for PC is diabetes, which is one of the various components that constitute MetS (13). According to UK Biobank data, the PC risk was increased in people with MetS (HR = 1.31, 95%CI 1.09–1.56), hyperglycemia (HR = 1.60, 95%CI 1.31–1.97), and abdominal obesity (HR = 1.24, 95%CI 1.02–1.50). However, these two last MetS components (central obesity and hyperglycemia) seem to exhibit an independent connection in increasing the risk of PC (12).

The present study indicated a correlation between MetS and the risk of PC. The hypothesis of this study was supported by the two case-control studies that were part of the systematic review. Low degrees of study heterogeneity were observed, however. Through subgroup analyses, the cause of heterogeneity was identified, and we came to the conclusion that among MetS patients, the risk of developing PC was higher in women than in men. This observation was consistent with the findings of one of the previous studies (25).

Moreover, a summary of each MetS component’s impact on PC risk was produced. According to the majority of studies (26, 27), PC risk is correlated with hypertension, hyperglycemia, low HDL-c levels, and particularly with hyperglycemia.

There has been extensive research on the pathogenesis of PC in diabetes mellitus or hyperglycemia. PC cells multiplied and invaded as a result of p38 MAPK elicited by high glucose levels. Additionally, P38 MAPK was also activated as a result of cellular stress and inflammatory conditions, which could control metastasis, apoptosis, and cell proliferation. PC cell proliferation and development occurred as a result of heightened paracrine effects of inflammatory cytokines (such as IL-6) and VEGF, which were mediated by P38 MAPK. Moreover, elevated hyperglycemia *via RET* (a proto-oncogene that encodes a receptor tyrosine kinase for members of the glial cell line-derived neurotrophic factor family of extracellular signaling molecules) can boost PC cell invasion and proliferation (18). Meta-analyses had also shown that dietary cholesterol might be linked to a higher risk of PC (28), which was confirmed by the results of this study. Surprisingly, obesity and hypertriglyceridemia were not associated with PC in the meta-analysis. Previous studies also revealed that there is an increased risk of developing cancer due to obesity (17, 29–31), contradictory to the outcomes of this study. Evidence suggested that the development and progression of PC were caused by an increase in various hormones in obese people, including insulin, adipokines, and resistin (18). Resistin is an adipocyte-secreting hormone involved in insulin resistance and inflammation. It has the ability to affect the progression of the PC. In patients with pancreatic ductal adenocarcinoma, it was considered a negative independent prognostic factor for relapse-free survival (32). Therefore, we speculate that the possible reason for this is insulin resistance and/or low HDL-c levels in most obese individuals, which can increase the cancer risk. Moreover, as per the outcomes of a meta-analysis performed in 2012, the body mass index and central obesity are linked to an average RR of 1.10 for a five-unit rise in the occurrence of PC (33). This correlation applies to African Americans (34) but not to residents of Lithuania (35) or Singapore’s Chinese nonsmoking population (36). Asians comprised the majority of the ethnicities examined in the studies used in the meta-analyses conducted in this research. The European Australasian (RR: 1.18, 95%CI 1.09–1.27) and North American (RR: 1.07, 95%CI 1.03–1.11) populations, however, showed favorable relationships between MetS and PC (37). These outcomes can be explained based on different study methodologies and variations, for example, socioeconomic, genotypic, and environmental aspects of these diverse groups.

Elevated triglyceride levels and reduced HDL-c are the components of MetS. Previous studies on dyslipidemia and the risk of PC produced controversial results (36, 38, 39). In the present study, no evidence of increased risk of developing PC due to high triglyceride levels was obtained.

MetS is reversible. In patients with MetS, and the lifestyle-modification intervention was successful. It resulted in easing the condition and decreasing the severity of associated abnormalities (triglycerides, waist size, systolic and diastolic blood pressure, and fasting blood glucose) (39). Previous results also suggested that MetS could be a risk factor for PC that is modifiable (22). The connection between MetS and the risk of PC may be explained by various molecular pathways. First, insulin resistance is a significant contributor to the pathophysiology of MetS. Elevated insulin levels, as well as modulation of insulin-like growth factors-1 and -2, may contribute to PC by boosting cell proliferation and angiogenesis while inhibiting cell death (40–42). Moreover, visceral adipose tissue has a high metabolic rate and secretes a variety of cytokines that promote inflammation (41, 42). Chroniclow-grade inflammation, including these cytokines, may increase the risk of PC by increased production of reactive oxygen species and cell cycle rates, thus attenuating tumor suppressor activity (42, 43). Finally, MetS have been linked to the altered composition of gut microbiota, decreased microbial diversity, and decreased gene richness, all of which are crucial for carcinogenesis and tumorigenesis (43, 44). Therefore, it can be concluded that preventing or recovering from MetS might reduce the risk of developing PC.

However, this meta-analysis has some limitations. First, like any other meta-analysis, residual confounding from the original studies cannot be eliminated. After correcting for the majority of significant confounding factors, residual or unknown confounders may persist. Because each trial was adjusted for a unique set of variables, meta-analyses may have been heterogeneous. Second, the comprehensiveness of this study was limited by the relatively small number of pertinent publications, which precluded analyses for other relevant characteristics, including age and ethnicity. Third, the metabolic components were not directly assessed using the same technique, which may result in high heterogeneity between studies. However, the sensitivity analysis and subgroup analysis showed the robustness of our outcomes.

In conclusion, our meta-analysis revealed that MetS showed a remarkable correlation with a high risk of developing PC in both genders, with a higher risk in females as compared to males. Low HDL-c levels or hyperglycemia may be primarily responsible for the higher risk of PC in individuals with MetS. However, obesity and hypertriglyceridemia do not increase the risk of PC.

## Data availability statement

The original contributions presented in the study are included in the article/**Supplementary Materials**. Further inquiries can be directed to the corresponding author.

## Author contributions

All authors contributed to the study’s conception and design. LZ was in charge of material preparation, data collection, and analysis. SL wrote the first draft of the paper. JFL contributed to the writing, revision, and review of the manuscript. GT reviewed the manuscript. All authors contributed to the article and approved the submitted version.
