# Supplementary material for: Correlation between pancreatic cancer and metabolic syndrome: A systematic review and meta-analysis
Source: Front Endocrinol (Lausanne). 2023 Apr 11;14:1116582. doi: 10.3389/fendo.2023.1116582 (PMC10126301; doi:10.3389/fendo.2023.1116582)
Supplement: Supplementary file 1 [file Table_1.docx]

| Obesity | RR_UL | - | - | 1.5 | 2.05 | 1.09 | 1.32 | 1.63 |
| --- | --- | --- | --- | --- | --- | --- | --- | --- |
|  | RR_LL | - | - | 1.02 | 0.35 | 0.79 | 0.87 | 1.12 |
|  | RR | - | - | 1.24 | 0.94 | 0.93 | 1.07 | 1.35 |
| hypertriglyceridemia | RR_UL | - | - | 1.15 | 1.47 | - | 1.1 | 1.28 |
|  | RR_LL | - | - | 0.77 | 0.22 | - | 0.78 | 0.88 |
|  | RR | - | - | 0.94 | 0.56 | - | 0.92 | 1.06 |
| low HDL-c level | RR_UL | - | - | 1.52 | 3.32 | - | 1.47 | 1.49 |
|  | RR_LL | - | - | 0.97 | 0.53 | - | 1.06 | 1.01 |
|  | RR | - | - | 1.21 | 1.32 | - | 1.25 | 1.23 |
| hyperglycemia | RR_UL | - | - | 1.97 | 2.21 | - | 1.69 | 2.06 |
|  | RR_LL | - | - | 1.31 | 0.68 | - | 1.34 | 1.39 |
|  | RR | - | - | 1.6 | 1.23 | - | 1.51 | 1.69 |
| hypertension | RR_UL | - | - | 1.42 | 1.5 | - | 1.19 | 1.44 |
|  | RR_LL | - | - | 0.85 | 0.53 | - | 0.98 | 0.98 |
|  | RR | - | - | 1.1 | 0.89 | - | 1.08 | 1.19 |
| Female | RR_UL | 2.26 | 3.45 | 2.02 | - | 2.32 | - | 2.25 |
|  | RR_LL | 0.87 | 0.94 | 1.2 | - | 1.3 | - | 1.27 |
|  | RR | 1.45 | 1.8 | 1.55 | - | 1.74 | - | 1.69 |
| Male | RR_UL | 2.66 | 1.22 | 1.58 | - | 1.44 | - | 1.71 |
|  | RR_LL | 1.14 | 0.07 | 0.98 | - | 0.89 | - | 1.07 |
|  | RR | 1.78 | 0.28 | 1.24 | - | 1.13 | - | 1.32 |
| All | RR_UL | 2.18 | 4.86 | 1.56 | 1.81 | 1.61 | 1.28 | 1.79 |
|  | RR_LL | 1.17 | 0.13 | 1.09 | 1.19 | 1.12 | 1.16 | 1.22 |
|  | RR | 1.62 | 0.8 | 1.31 | 1.47 | 1.34 | 1.22 | 1.48 |
| MetS（-） | | - | 48 | 347 | 192 | 714 | 4276 | - |
| MetS（+） | | 43 | 17 | 191 | 189 | 172 | 3734 | - |
| Sample size n total | | 43 | 65 | 565 | 381 | 886 | 8010 | 478 |
| Author | | Antonio Russo^[20]^ | Manami Inoue^[15]^ | Bin Xia^[12]^ | Sung Keun Park^[14]^ | Hye Soo Chung^[21]^ | Joo-Hyun Park^[22]^ | Joseph A^[24]^ |

TableS1. Original data from the articlesincluded in the meta-analysis
